# My personal highlights of ESMO 2016

**DOI:** 10.1007/s12254-017-0314-8

**Published:** 2017-02-08

**Authors:** M. Dediu, A. Gerger, N. Zojer, R. Bartsch

**Affiliations:** 1 Department of Oncology, Sanador Hospital, Bucharest, Romania; 2 Department of Medicine, Division of Oncology, Medical University of Graz, Graz, Austria; 3 Department of Haematology and Medical Oncology, Wilhelminenspital Vienna, Vienna, Austria; 4 Department of Medicine 1, Division of Oncology, Medical University of Vienna, Waehringer Guertel 18-20, Vienna, 1090 Austria

**Keywords:** ESMO 2016 meeting, Highlights, Immunotherapy, Niraparib, Ribociclib

## Abstract

Results of several clinically relevant studies were presented at the 2016 Annual Meeting of the European Society of Medical Oncology (ESMO). This article summerizes the personal highlights of three medical oncologists in their respective areas of expertise.

The 2016 Annual Meeting of the European Society of Medical Oncology (ESMO) was characterized by an abundance of presentations of novel and potentially practice-changing clinical trial results never seen before at a European cancer conference. This clearly underlines the importance of this meeting among the plethora of other oncology congresses. Within this huge amount of data, two main trends were observed: Immunotherapy with immune checkpoint modulators is still at the very center of scientific interest with further treatment individualization being a second main emphasis.

This short review reflects the personal highlights of three experts in medical oncology in their respective fields of expertise.

## Dr. Dediu:

My personal highlights of ESMO 2016:

- Paradigm-changing data: Ipilimumab was superior to placebo [1] as adjuvant therapy of malignant melanoma while pembrolizumab was better than chemotherapy as first-line therapy in non-small-cell lung cancer (NSCLC) with ≥50% PD-L1 expression without *EGFR* mutation or *ALK* translocation [2], for both in terms of overall survival.
- Data-confirming important previous advances: Addition of the CDK4/6 inhibitor ribociclib to the aromatase-inhibitor (AI) letrozole as first-line therapy in estrogen-receptor (ER)-positive HER2-negative patients with advanced breast cancer yielded superior progression-free survival (PFS) data over endocrine therapy alone [3]. Niraparib, a novel PARP inhibitor, prolonged PFS when used as maintenance therapy in platinum-sensitive ovarian cancer irrespective of the presence or absence of *BRCA* germline mutations [4]. Atezolizumab, a monoclonal antibody targeting PD-L1, was successfully tested as second- and third-line therapy in NSCLC [5] while other immune checkpoint modulators such as pembrolizumab [6] and nivolumab [7] showed promising results in advanced urothelial cancer.
- Promising data that should await further confirmation before being translated into the clinical routine setting: Sunitinib was superior to placebo as adjuvant treatment of renal cell cancer (RCC) [8]; furthermore, cabozantinib was superior to sunitinib as first-line therapy of metastatic RCC [9]. Finally, the anti-estrogen fulvestrant was superior to the AI anastrozole as first-line therapy in ER-positive metastatic breast cancer although this benefit was apparently restricted to patients with visceral metastases [10].

## Dr. Gerger:

Colorectal cancer “sidedness”:

Colorectal cancer is a heterogeneous disease. In contrast to the left-sided colon, which derives from the embryonic hindgut, the right-sided colon originates from the midgut.

Primary tumors arising from different regions of the colon are molecularly and clinically distinct. Left-sided tumors more frequently possess a molecular profile of an EGFR inhibitor-sensitive phenotype, which is reflected in *EGFR/ERB-B2* and epiregulin amplification; conversely, right-sided tumors show more frequently *BRAF* mutations and microsatellite instability. These molecular differences manifest in different clinical behavior, with right-sided tumors harboring a worse prognosis. At this year’s ESMO conference in Copenhagen, the predictive value of colon cancer sidedness was comprehensively discussed including retrospective analyses of the anti-VEGF versus anti-EGFR head-to-head trials FIRE-3, PEAK, and CALGB80405 [11]. In summary, in first-line therapy of patients with left-sided *RAS* wild-type tumors, a combination therapy consisting of an EGFR antibody with chemotherapy is recommended. In patients with right-sided *RAS* wild-type tumors, there is presently no proven benefit of an EGFR antibody compared with bevacizumab. Therefore, a bevacizumab plus chemotherapy combination is recommended.

## Dr. Zojer:

My personal highlights of ESMO 2016:

Apart from the seminal results of the KEYNOTE-024 and CheckMate-026 lung cancer trials, the following two presentations seem to have practice-changing impact: Mirza et al. [4] reported results from a randomized double-blind phase 3 trial of maintenance therapy with the PARP inhibitor niraparib versus placebo (2:1) in patients with platinum-sensitive recurrent ovarian cancer (ENGOT-OV16/NOVA trial). Overall, 553 patients were enrolled after response to platinum chemotherapy, 203 with a germline *BRCA* mutation and 350 patients without such a mutation. Interestingly, niraparib maintenance improved outcome in terms of PFS not only in the patient cohort with *BRCA* mutation (PFS, 21.0 months versus 5.5 months; *p* < 0.0001) but also in the *BRCA* wild-type subset (PFS, 9.3 months versus 3.9 months, *p* < 0.0001). Although overall outcome was better in the mutant subset, these results seem to indicate that PARP inhibition is an effective treatment modality for ovarian cancer independent of germline *BRCA* mutation status. Important results were also reported on the adjuvant treatment of stage III melanoma. Eggermont et al. [1] showed that ipilimumab not only prolonged relapse-free survival in this setting, but also led to an improvement in terms of overall survival compared with placebo (5-year overall survival 65.4% versus 54.4%; *p* = 0.001; EORTC 18071 trial).
